# Democratic Thwarting of Majority Rule in Opinion Dynamics: 1. Unavowed Prejudices Versus Contrarians

**DOI:** 10.3390/e27030306

**Published:** 2025-03-14

**Authors:** Serge Galam

**Affiliations:** CEVIPOF—Centre for Political Research, Sciences Po and CNRS, 1, Place Saint Thomas d’Aquin, 75007 Paris, France; serge.galam@sciencespo.fr

**Keywords:** opinion dynamics, democratic balance, thwarting, prejudices, contrarians, tipping points, attractors, sociophysics

## Abstract

I study the conditions under which the democratic dynamics of a public debate drives a minority-to-majority transition. A landscape of the opinion dynamics is thus built using the Galam Majority Model (GMM) in a 3-dimensional parameter space for three different sizes, r=2,3,4, of local discussion groups. The related parameters are $\left(p_{0},k,x\right)$, the respective proportions of initial agents supporting opinion A, unavowed tie prejudices breaking in favor of opinion A, and contrarians. Combining *k* and *x* yields unexpected and counterintuitive results. In most of the landscape the final outcome is predetermined, with a single-attractor dynamics, independent of the initial support for the competing opinions. Large domains of (k,x) values are found to lead an initial minority to turn into a majority democratically without any external influence. A new alternating regime is also unveiled in narrow ranges of extreme proportions of contrarians. The findings indicate that the expected democratic character of free opinion dynamics is indeed rarely satisfied. The actual values of (k,x) are found to be instrumental to predetermining the final winning opinion independently of $p_{0}$. Therefore, the conflicting challenge for the predetermined opinion to lose is to modify these values appropriately to become the winner. However, developing a model which could help in manipulating public opinion raises ethical questions. This issue is discussed in the Conclusions.

## 1. Introduction

Today, in most democratic countries the demand for more direct democracy is growing substantially. In particular, launching public debates and referendums to address major contemporary societal issues is seen as paramount to ensuring subsequent democratic choices, as opposed to decisions taken by political decision-makers, who are often perceived as being disconnected from the reality experienced by citizens [1].

A follow-up collective majority voting then achieves the democratic process of selection of appropriate policies to deal with the essential topics contemporary societies are facing. With majority-rule voting between two competing choices, in principle, one ballot difference is enough to determine the winner of a binary vote. Clearly, that never happens for large-scale voting. Nevertheless, the outcome can be very tight, as observed with hung elections [2].

In this paper, I question the validity of what I define as the “belief” that an open public debate brings out the choice that is supported by the majority, i.e., more than half of the community concerned by the issue at stake.

To be precise, I do not question the democratic nature of majority-rule voting, I claim that the debate taking place prior to the vote is likely to modify the initial majority of individual choices through invisible and unconscious biases, most often favorable to the initial minority but not always. I thus focus on unveiling the conditions under which a public debate twists “naturally” the initial majority–minority balance by implementing a minority-to-majority transition. When more than two choices are competing, the fairness and flaws of majority voting could be also at stake, as discussed a long time ago along a different logic [3].

The present work subscribes to the emerging and active field of sociophysics [4,5,6,7]. Sociophysics explores and tackles social, political, and psychological phenomena by adopting a physicist-like approach [8,9]. The goal is not to substitute for the social sciences but to create a new hard science by itself [10,11,12,13,14,15,16,17].

Thanks to its universal features, sociophysics can deal with a rather large spectrum of different issues [18,19,20,21,22,23,24,25,26,27,28,29,30,31,32,33,34,35,36,37,38,39,40,41,42,43,44,45]. Among them, the study of opinion dynamics has been particularly prominent [46,47,48,49,50,51,52,53,54,55,56,57,58,59,60,61]. Many papers address binary variables [62,63,64,65,66,67,68,69,70,71,72,73,74,75,76,77,78,79,80], with a few addressing three or more discrete opinions [81,82,83,84,85,86,87,88,89]. Continuous variables have also been considered [90,91,92].

Indeed, the Galam Majority Model (GMM) has already highlighted a phenomenon of a minority-to-majority transition, which goes unnoticed, being unconscious and invisible to the involved agents [93,94,95]. In particular, the GMM has unveiled the drastic biasing effect of some psychological traits, including tie-breaking prejudice [95], contrarian behavior [2], and stubbornness [96,97].

In the following, *k* denotes the probability that at a tie, unavowed prejudices break the local symmetry in favor of opinion A. The probability is (1−k) in favor of opinion B. The proportion of contrarians is denoted *x* and $p_{0}$ is the proportion of initial agents supporting opinion A. These three traits have been shown to produce different types of polarization: respectively, unanimity, coexistence, and rigidity [98].

I then explore the opinion dynamics landscape of the GMM in the full 3-dimensional parameter space $\left(p_{0},k,x\right)$. Three different sizes, r=2,3,4, are used for the local update groups. The focus is on the minority-to-majority transition in the $\left(p_{0},k,x\right)$ parameter space. The individual traits associated with *k* and *x* are invisible to agents.

Previous studies have shown that tie-breaking prejudices and contrarians have opposite effects [2,95]. On one hand, tie-breaking prejudices implement the phenomenon of minority spreading with two different asymmetric tipping points $p_{T}$ for opinion A and $\left(1-p_{T}\right)$ for opinion B [95] between two attractors. On the other hand, contrarians reduce the gap between the two attractors, with them eventually merging at fifty percent at rather low values of *x* [2].

However, combining *k* and *x* was found to yield unexpected and counterintuitive results [99]. The investigation was restricted to the subspace (k,x<0.50) for update groups of size 4. Yet, the findings allowed me to propose an alternative novel scheme to block the propagation of fake news without banning it but using sequestration instead.

Here, I extend the investigation to the full subspace (k,x) using the three different sizes r=2,3,4 for the local update groups. For same range of values, the mixing of *k* and *x* is shown to yield an unexpected reversal of their resulting net impact on the opinion dynamics. Now, contrarians amplify the bias associated with tie-breaking prejudices as opposed to restoring a balance between both competing opinions as before. In addition, at high proportions of contrarians, the interplay with tie-breaking prejudices creates a new unknown regime of alternating polarization.

The results allow for the identification of the ranges of values of (k,x) where an initial support $p_{0}<\frac{1}{2}$ ends up as $p_{n}>\frac{1}{2}$ after *n* successive updates of individual opinions, thus democratically turning the minority to the majority without any deliberate and conscious manipulation, neither internal nor external. The findings indicate that the expected democratic nature of free opinion dynamics is rarely met. Often, an initial minority of agents turn to their side a large fraction of agents who initially support the opposite majority opinion.

On this basis, noting that the final outcome of a debate is often predetermined, it appears that the only available option for the supporters of the predetermined defeated opinion would be trying to modify the actual values of the pair (k,x) to reach a location which is beneficial to that opinion in place of the other one.

Nevertheless, identifying the means to implement changes in *k* or and *x* is out of the scope of the present paper. Moreover, such a strategy raises ethical issues about developing a model which could help in manipulating public opinion. I address this issue in the Conclusions.

The rest of the paper is organized as follows: Section 2 reviews the spontaneous thwarting of democratic global balance in homogeneous populations for discussion groups of sizes r=2,3,4. Section 3 considers heterogeneous agents with the introduction of contrarians. The combined effect of contrarians and tie-breaking prejudice is investigated in Section 4. The occurrence of a new unexpected regime of stationary alternating polarization is discussed in Section 5. Section 6 contains a summary of the main results with a word of caution about the responsibility of developing a model which could eventually lead to the manipulation of opinion dynamics.

## 2. Spontaneous Thwarting of Democratic Global Balance in Homogeneous Populations

The Galam Majority Model (GMM) of opinion dynamics iterates local majority rule to small groups of agents who are reshuffled after each update.

For a homogeneous population of rational agents, a democratic balance is obtained with a tipping point located at 50% and two attractors at 0% and 100%. Starting from a population divided into agents supporting two parties A and B with respective supports $p_{0}$ and $\left(1-p_{0}\right)$, a first cycle of local updates using discussion groups of size *r* yields new supports $p_{1}$ and $\left(1-p_{1}\right)$.

Using odd-sized groups guarantees the winning of an initial majority $p_{0}>0.5$ with $p_{0}<p_{1}<p_{2}<\ldots$ On the contrary an initial minority $p_{0}<0.5$ loses the debate with $p_{0}>p_{1}>p_{2}<\ldots$ A number *n* of cycles is required to reach one of the two attractors. The value of *n* is a function of $p_{0}$ and *r*; it is always less than 12 in the range $p_{0}<0.49$ and $p_{0}>0.51$.

However, a first thwarting of the global democratic balance occurs in the case of even-sized groups. At a tie, assuming that silent tie-breaking prejudices select A with probability *k* and B with probability (1−k), the 50% tipping point splits into two tipping points, located, respectively, at high and low values.

One opinion can now spontaneously become the majority, even when starting as a minority, and subsequently an initial opposite majority shrinks to a minority via repeated open-mind discussions among small groups of rational agents. The update equations for groups of size 2, 3, and 4 are

(1) $$p_{i+1,2,k}=p_{i,2,k}^{2}+2kp_{i,2,k}\left(1-p_{i,2,k}\right),$$

(2) $$p_{i+1,3}=p_{i,3}^{3}+3p_{i,3}^{2}\left(1-p_{i,3}\right),$$

(3) $$p_{i+1,4,k}=p_{i,4,k}^{4}+4p_{i,4,k}^{3}\left(1-p_{i,4,k}\right)+6kp_{i,4,k}^{2}{\left(1-p_{i,4,k}\right)}^{2},$$

where $p_{i+1,r,k}$ ($p_{i+1,r}$) is the new proportion of support A after one cycle of updates from a proportion $p_{i,r,k}$ ($p_{i+1,r}$) using groups of size *r*; here, with r=2,3,4.

Odd-sized groups have no tie and are always independent of *k*. Accordingly, from here on, when the update is independent of *k* or $k=\frac{1}{2}$ (balanced effect of prejudices), the parameter *k* is not included in the indices defining *p*.

The three update equations yield the same two fixed points, $p_{B}=0$ and $p_{A}=1$, with an additional tipping point for the last two, respectively, $p_{T}=\frac{1}{2}$ and $p_{T,4,k}=\frac{1-6k+\sqrt{13-36k+36k^{2}}}{6(1-2k)}$, which in turn makes $p_{B}$ and $p_{A}$ attractors. For the first case (r=2), $k<\frac{1}{2}$ makes $p_{B}$ a tipping point and $p_{A}$ an attractor, with the opposite for $k>\frac{1}{2}$. For $k=\frac{1}{2}$, the update has no effect, with $p_{i+1,2,k=1/2}=p_{i,2,k=1/2}$. In addition, $p_{T,4,k=0}\approx 0.77$, $p_{T,4,k=1/2}=\frac{1}{2}$, and $p_{T,4,k=1}\approx 0.23$. This last case illustrates the phenomenon of minority spreading, with any $p_{0}>0.23$ resulting in A winning.

The above cases show how hidden prejudices thwart the democratic global balance of a dynamics obeying local majority rule in a homogeneous population of rational agents. Only the case r=3 ensures a democratic balance due to the absence of ties in the discussion groups.

**Figure 1:**
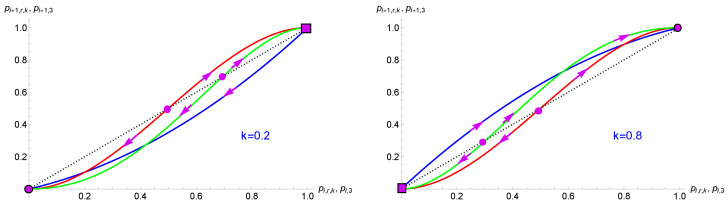
Update curves $p_{i+1,r,k}$ as a function of $p_{i,r,k}$ for r=2 (blue curves) and r=4 (green curves) at k=0.2 (**left**) and k=0.80 (**right**). Update curve for $p_{i+1,3}$ (r = 3) as a function of $p_{i,3}$ is also shown (red curves). The dotted lines represent the diagonal $p_{i+1}=p_{i}$. Arrows indicate update directions, solid circles indicate tipping points, solid circles with a border indicate attractors and solid squares with a border indicate either attractors or tipping points depending on *r* and *k*. Only r=3 yields a democratic balance, with $p_{T}=\frac{1}{2}$.

Figure 1 shows the update curves $p_{i+1,r,k}$ as a function of $p_{i,r,k}$ for r=2 and r=4 at k=0.2 and k=0.80. The curve $p_{i+1,r=3}$ as a function of $p_{i,r=3}$ is also shown. The directions of the update flows are also indicated with the tipping points and attractors.

## 3. Heterogeneous Agents: The Contrarian Thwarting

I now introduce a proportion *x* of contrarian agents with a proportion (1−x) of rational agents. A contrarian agent is identical to a rational agent beside the fact that once the discussion is over, they do no follow the majority opinion but shift to the opposite of whatever is the majority opinion. Contrarians are not identifiable. The associated update equation for groups of size *r* is

(4) $$\begin{array}{ccc} p_{i+1,r,x} & = & \left(1-x\right)p_{i+1,r,x}+x\left\{1-p_{i+1,r,x}\right\},\\ & = & \left(1-2x\right)p_{i+1,r,x}+x, \end{array}$$

where $p_{i+1,r,x}$ has been defined above. There is no prejudice effect for odd sizes and even sizes with $k=\frac{1}{2}$.

### 3.1. Size 2

I start studying the contrarian impact on the landscape of a democratic dynamics of opinion by considering discussion groups of size 2. The update Equation (4) becomes

(5) $$p_{i+1,2,x}=\left(1-2x\right)p_{i,2,x}+x,$$

with the unique attractor $p_{A,B,2}=\frac{1}{2}$ provided x≠0. At x=0, $p_{i+1,2,x=0}=p_{i,2,x=0}.$ For whatever initial respective support there is for A and B, even a handful of contrarians drives the dynamics towards fifty–fifty creating a hung election outcome, which is non-democratic. Moreover, any actual measure does not yield the expected fifty–fifty due to incompressible statistical fluctuations but an outcome very close to 0.50. Accordingly, one opinion wins with a “wrong”. The winner is thus de facto the result of chance, which could lead the loser to claim that the outcome is the result of fraud. An exact counting, which is never feasible for a large number of ballots, would produce no winner, with exactly 50% for each choice [2].

### 3.2. Size 3

For groups of size 3, the associated update equation can be written as

(6) $$p_{i+1,3,x}=\left(1-2x\right)\left\{p_{i,3,x}^{3}+3p_{i,3,x}^{2}\left(1-p_{i,3,x}\right)\right\}+x,$$

whose dynamics is driven by the two attractors,

(7) $$p_{B(A),3,x}=\frac{1-2x\mp \sqrt{1-8x+12x^{2}}}{2(1-2x)},$$

and the tipping point $p_{T}=\frac{1}{2}$.

**Figure 2:**
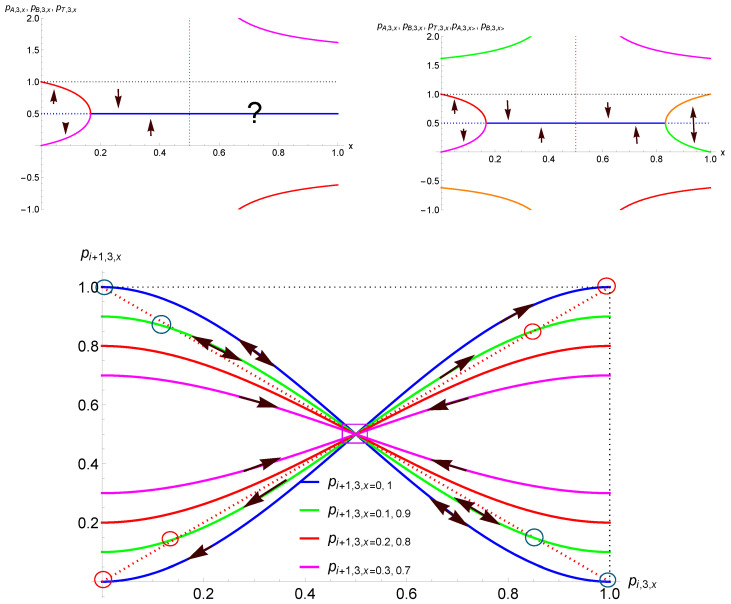
Attractors and tipping points yielded by Equation (6) are shown on the upper left side for r=3 as a function of the proportion *x* of contrarians. Only the values between 0 and 1 are meaningful. Solid lines (in red, magenta, blue) are attractors while tipping points (in dotted blue) exist only for $x<\frac{1}{6}\approx 0.17$. When $x>\frac{1}{6}$, one unique attractor drives the dynamics toward perfect equality. For $x>\frac{1}{2}$, with more than half the population being contrarians, alternating dynamics is expected. The upper right side shows the completed dynamics with a single-attractor dynamics for $\frac{1}{6}<x<\frac{5}{6}$ and a dynamics with two alternating attractors when $x>\frac{5}{6}$. Update curves $p_{i+1,3,x}$ as a function of $p_{i,3,x}$ for x=0,0.10,0.20,0.30 and x=0.70,0.80,0.90,1 are shown in the lower part. The dotted red lines represent the diagonals $p_{i+1}=p_{i}$ and $p_{i+1}=1-p_{i}$. Arrows indicate update directions, red circles indicate attractors, and blue circles indicate alternating attractors. The $p_{T}=\frac{1}{2}$ square in the middle is either a tipping point, when $x<\frac{1}{6}$ and $x>\frac{5}{6}$, or an attractor when $\frac{1}{6}<x<\frac{5}{6}$. The lower part shows $p_{i+1,3,x}$ as a function of $p_{i,3,x}$ for x=0,0.10,0.20,0.30,0.70,0.80,0.90,1. Arrows indicate the direction of the updates. Double arrows signal an alternating dynamics.

However, $p_{B,3,x}$ and $p_{A,3,x}$ are defined only as long as $1-8x+12x^{2}\geq 0$ and $0\leq p_{B,3,x},p_{A,3,x}\leq 1$, which is satisfied in the range $x\leq \frac{1}{6}\approx 0.17$. At this stage, the dynamics is globally democratic with the spreading of the initial majority. The contrarian effect prevents unanimity being reached. In addition, at $x=\frac{1}{6}$ the two attractors merge at the tipping point, turning it into the unique attractor of the dynamics. When $x\geq \frac{1}{6}$, the above democratic dynamics is thus suddenly turned upside down, with a single-attractor dynamics at $p_{T}=\frac{1}{2}$. Any initial conditions end up at 50%, breaking the previous democratic balance. These results are exhibited on the left side of Figure 2.

The update curves $p_{i+1,3,x}$ as a function of $p_{i,3,x}$ for x=0,0.10,0.20,0.30 are shown in the lower part of Figure 2. The directions of the update flows are also indicated with the tipping points and attractors.

**Figure 3:**
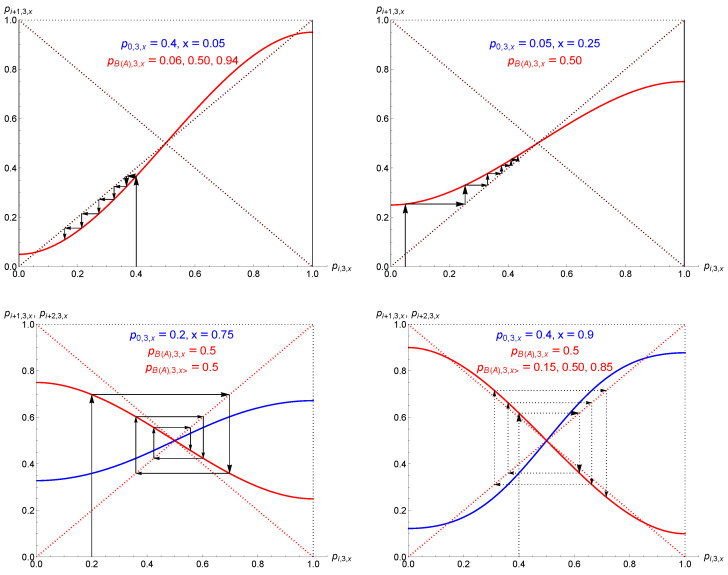
The upper left part shows the update Equation (6) for x=0.105, which has a tipping point at 0.50 with two attractors at 0.06 and 0.94. The dynamics is illustrated from $p_{0,3,x}=0.40$. The upper right part shows the case x=0.25, which is a single-attractor dynamics with one attractor at 0.50. The dynamics is illustrated from $p_{0,3,x}=0.05$. The lower part exhibits both updates $p_{i+1,3,x}$ in red and $p_{i+2,3,x}$ in blue as function of $p_{i,3,x}$. The left side shows the case x=0.75, which is an alternating single-attractor dynamics with one attractor at 0.50. The dynamics is illustrated from $p_{0,3,x}=0.20$. The right side shows the case x=0.90, which is an alternating dynamics with a tipping point at 0.50 and two alternating attractors at 0.15 and 0.85. The dynamics is illustrated from $p_{0,3,x}=0.40$.

In addition, I note that in the range $\frac{1}{2}<x\leq 1$ the dynamics is peculiar, with more than half of the community being contrarian. Although such a situation could sound socially awkward, it is interesting to investigate the related dynamics. The upper right part of Figure 2 and the lower part of Figure 3 exhibit a symmetry between the ranges $x<\frac{1}{2}$ and $x>\frac{1}{2}$, with an alternating update performed for the second case. This effect is also observed in the lower part of Figure 2 between the update curves for, respectively, x=0,0.10,0.20,0.30 and x=0.70,0.80,0.90,1.

This statement is proved by solving the equation $p_{i+2,3,x}=p_{i,3,x}$ to determine all its nine solutions. In addition to the above two attractors $p_{B(A),3,x}$ and tipping point $p_{T}=\frac{1}{2}$, four solutions are found to be complex, and the last two can be written as

(8) $$p_{B(A),3,x>}=\frac{1-2x\pm \sqrt{5-16x+12x^{2}}}{2(1-2x)},$$

which become identical to $p_{B(A),3,x}$ by substituting (1−x) for *x*. Similarly to $p_{B(A),3,x}$ being valid in the range 0≤x≤1/6, the attractors $p_{B(A),3,x>}$ are valid only in the range 5/6≤x≤1.

Therefore, the function $p_{i+2,3,x}$ exhibits a single-attractor dynamics located at $\frac{1}{2}$ as a function of $p_{i,3,x}$ for 1/2≤x≤5/6, as seen from the lower left part of Figure 3. The lower right part shows a threshold dynamics, as expected for 5/6≤x≤1. It is worth noting that in this case the associated dynamics alternates between the two attractors $p_{B(A),3,x>}$ for $p_{i+1,3,x}$ as a function of $p_{i,3,x}$. They are alternating attractors. These attractors can also be obtained by solving $p_{i+1,3,x}=1-p_{i,3,x}$ due to the symmetry of the update Equation (6).

### 3.3. Size 4

In the case of size 4, to study the impact of contrarian agents and avoid possible confusion with the prejudice effect, I set $k=\frac{1}{2}$ as above for size 2. The update Equation (4) can then be written as

(9) $$p_{i+1,4,x}=\left(1-2x\right)\left\{p_{i,4,x}^{4}+4p_{i,4,x}^{3}\left(1-p_{i,4,x}\right)+3p_{i,4,x}^{2}{\left(1-p_{i,4,x}\right)}^{2}\right\}+x,$$

which is found to be identical to Equation (6).

The dynamics is thus driven by the same landscape as for r=3, which is shown in Figure 2 and Figure 3.

## 4. Combined Thwarting Effect of Prejudices and Contrarians

The spontaneous thwarting created by tie-breaking prejudice at even sizes sizes favors one choice over the other. In contrast, the contrarian effect tends to first smooth and then suppress any difference between the two choices. Yet, both effects break the democratic balance associated with the aggregated initial majority.

I now investigate the combined effect of simultaneous prejudice breaking and contrarians for the two cases r=2 and r=4. The case r=3 is not considered in this section since no prejudice effect occurs for odd sizes.

### 4.1. Size 2

When prejudice breaking is added to contrarians, the update Equation, Equation (5), becomes

(10) $$p_{i+1,2,k,x}=\left(1-2x\right)\left\{p_{i,2,k,x}^{2}+2kp_{i,2,k,x}\left(1-p_{i,2,k,x}\right)\right\}+x,$$

whose fixed points are given by

(11) $$p_{B(A),2,k,x}=\frac{1-2k+4kx\mp \sqrt{{\left(1-2k+4kx\right)}^{2}-4x\left(1-2k\right)\left(1-2x\right)}}{2(1-2k)(1-2x)},$$

with the term in the square root always being positive for 0≤k≤1 and 0≤x≤1.

**Figure 4:**
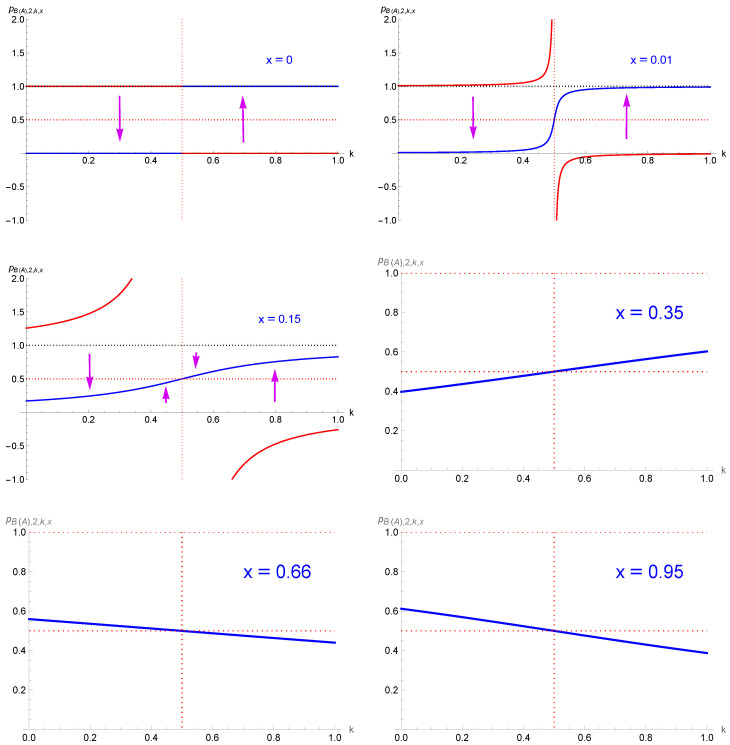
The upper left part of the figure shows the attractors and tipping points driving the dynamics as a function of *k* when x=0 for groups of size 2. Blue color lines indicate attractors. Red lines correspond to tipping points. Arrows shows the direction of update dynamics. The upper right part shows the effect of a tiny proportion of contrarians (x=0.01) turning the dynamics to single-attractor. The prejudice effect is simultaneously slightly reduced. A larger proportion of contrarians accentuates the reduction effect of prejudice, as seen in the middle left part with x=0.15. From x≈0.30 to x=1, $p_{B,2,k,x}$ is quasi-linear as a function of *k* for a given *x*, as illustrated in the middle right part and left and right lower part for x=0.35,0.65,0.95.

At x=0, the values of Section 2 are recovered with $p_{B(A),2,k,x=0}=0\left(1\right)$. However, as soon as x≠0, the fixed point $p_{A,2,k,x}$ is no longer valid, being outside the interval [0,1], as seen in Figure 4. In the range [0,1], the unique valid fixed point is thus $p_{B,2,k,x}$. It is located lower than $\frac{1}{2}$ in the range $0\leq k<\frac{1}{2}$ and higher than $\frac{1}{2}$ for $\frac{1}{2}<k\leq 1$. At $k=\frac{1}{2}$, $p_{B,2,k,x=1/2}=\frac{1}{2}$.

Moreover, $p_{B,2,k,x}$ is quasi-linear as a function of *k* for a given *x* from x≈0.30 to x=1, as illustrated in Figure 4. In addition, for $\frac{1}{2}\leq x\leq 1$, not much change occurs, with a slight variation in the associated slope. In the range $0<x<\frac{1}{2}$, $p_{B,2,k,x}$ is the attractor of the dynamics.

#### 4.1.1. Dynamics $x>\frac{1}{2}$: Part 1

For $x>\frac{1}{2}$, contrarians being in the majority makes the dynamics oscillate. In this range, the case r=3 reveals an alternating-tipping-point regime for $x>\frac{5}{6}$. This regime could be anticipated by symmetry from the tipping-point regime observed for $x<\frac{1}{6}$. In contrast, for r=2, there is no tipping-point regime for a low concentration of contrarians $x<\frac{1}{6}$. In addition, there is no symmetry between *x* and 1−x, as for r=3.

These two facts hint against the existence of an alternating-tipping-point regime at a high concentration of contrarians. However, solving the fixed-point equation $p_{i+1,2,k,x}=p_{i,2,k,x}$ of the double iteration reveals an unexpected behavior at a very high concentration of contrarians. For the fixed-point equation, being a polynomial of degree 4, two new fixed points,

(12) $$p_{B(A),2,k,x>}=\frac{-1-2k+4kx\mp \sqrt{-3-4k\left(1-k\right){\left(1-2x\right)}^{2}-4x\left(1-2x\right)}}{2(1-2k)(1-2x)},$$

are obtained in addition to $p_{B(A),2,k,x}$, also given by Equation (11). To be valid the additional fixed points must obey $0\leq p_{B(A),2,k,x>}\leq 1$ with $-3-4k\left(1-k\right){\left(1-2x\right)}^{2}-4x\left(1-2x\right)\geq 0$, which is the case for

(13) $$x>\frac{1-4k+4k^{2}+\sqrt{7-12k+12k^{2}}}{4(1-2k+2k^{2})},$$

where $7-12k+12k^{2}\geq 4$ for 0≤k≤1.

**Figure 5:**
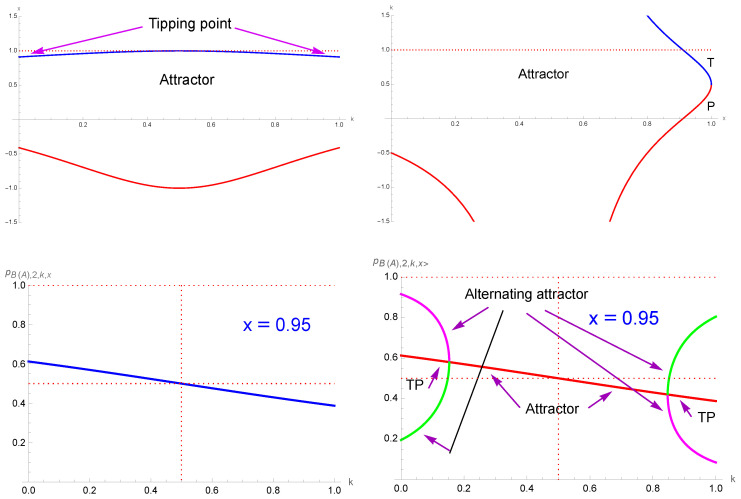

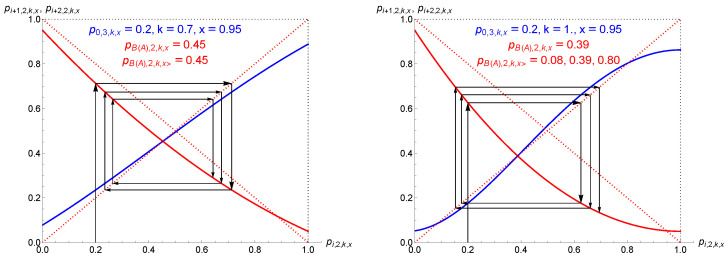
The upper part of the figure shows the domains with a single-attractor dynamics and an alternating-tipping-point dynamics for the update $p_{i+1,2,k,x}$. The left part shows them for *x* as a function of *k* and the right part for *k* as a function of *x* (TP = tipping point). The middle part shows the single fixed point $p_{B,2,k,x}$ for x=0.95 as a function of *k*; the left part when only the update $p_{i+1,2,k,x}$ is used; and the right part when the stability of the fixed point has been added using either $p_{i+2,2,k,x}$ or the derivative of $p_{i+1,2,k,x}$ with respect to $p_{i,2,k,x}$ at the fixed point. The lower part exhibits both $p_{i+1,2,k,x}$ and $p_{i+2,2,k,x}$ as a function of $p_{i+1,2,k,x}$ for x=0.95, with k=0.7 on the left and k=1 on the right. Arrows shows the evolution of $p_{i,2,k,x}=0.20$ for six successive updates for each case. Vertical ones show the effect of one update while horizontal ones show from where the next update takes place. Blue curves show $p_{i+1,3,x}$ and red curves show $p_{i+2,3,x}$ as a function of $p_{i,3,x}$.

Figure 5 shows that $p_{B(A),2,k,x>}$ exists only in a narrow range of very high values of *x* as a function of *k* with a minimum of $x=\frac{1+\sqrt{7}}{4}\approx 0.91$ at k=0 and k=1, with x=1 at $k=\frac{1}{2}$.

The domain of validity of the additional fixed points can also be defined for *k* as a function of *x* instead of *x* as a function of *k*, given by Equation (13). The related condition can be written as

(14) $$k<\frac{1}{2}-\frac{\sqrt{1-x^{2}}}{\left(1-2x\right)}\;\;\vee \;\;k>\frac{1}{2}+\frac{\sqrt{1-x^{2}}}{\left(1-2x\right)},$$

whose associated domain is also shown in Figure 5. The domain extension stays very narrow.

Implementing the above findings enriches the landscape diagram of the dynamics for $x>\frac{1}{2}$, as exhibited in the middle part of Figure 5. For every pair *k* and (1−k) there exists one range of $x>\frac{1+\sqrt{7}}{4}$ for which an alternating-tipping-point regime is activated, as seen in the left upper part of Figure 5. But for most values of *x* no alternating-tipping-point regime takes place, with instead one single-attractor dynamics. Only in the range $x>\frac{1+\sqrt{7}}{4}$ do two ranges of *k* exist, at low and large values, as given by Equation (14), for which an alternating-tipping-point dynamics is created, as seen in the upper right part of Figure 5.

#### 4.1.2. Dynamics $x>\frac{1}{2}$: Part 2

Alternatively, I can skip analyzing the double-update equation and instead investigate directly the nature of the unique fixed point $p_{B,2,k,x}$. If $p_{B,2,k,x}$ is stable, it is an attractor, whereas if it is unstable, it is a tipping point. Each regime is then determined by the value of the derivative of $p_{i+1,2,k,x}$ with respect to $p_{i,2,k,x}$ taken at the fixed point, which yields

(15) $$\lambda_{2,k,x}\equiv \frac{\partial p_{i+1,2,k,x}}{\partial p_{i,2,k,x}}{|}_{p_{B,2,k,x}}=1-\sqrt{{\left(1-2k\left(1-2x\right)\right)}^{2}-4x\left(1-2k\right)\left(1-2x\right)},$$

using Equations (10) and (11). The condition $-1<\lambda_{2,k,x}<1$ implies the stability of $p_{B,2,k,x}$, which is then an attractor. In contrast, $\lambda_{2,k,x}<-1$ or $\lambda_{2,k,x}>1$ makes $p_{B,2,k,x}$ unstable, thus becoming a tipping point. The domain associated with the tipping regime is determined by

(16) $$0\leq k<\frac{1}{2}\;\;\wedge \;\;\frac{1}{2}<k\leq 1\;\;\wedge \;\;\frac{1-4k+4k^{2}+\sqrt{7-12k+12k^{2}}}{4(1-2k+2k^{2})}<x\leq 1,$$

with $\lambda_{2,k,x}=1$ for $k=\frac{1}{2}$. In this case, $p_{B,2,k,x}$ is an attractor. These conditions can be recast as

(17) $$\frac{1+\sqrt{7}}{4}<x\leq 1\;\;\wedge \;\;\left\{0\leq k<\frac{1}{2}-\frac{\sqrt{1-x^{2}}}{\left(1-2x\right)}\;\;\vee \;\;\frac{1}{2}+\frac{\sqrt{1-x^{2}}}{\left(1-2x\right)}<k\leq 1\right\}.$$

As expected, Equations (16) and (17) are identical to Equations (14) and (13).

While this method is more direct for determining the domain of a tipping-point dynamics, it does not identify the two associated alternating attractors. The double-update equation is required to localize them.

### 4.2. Size 4

**Figure 6:**
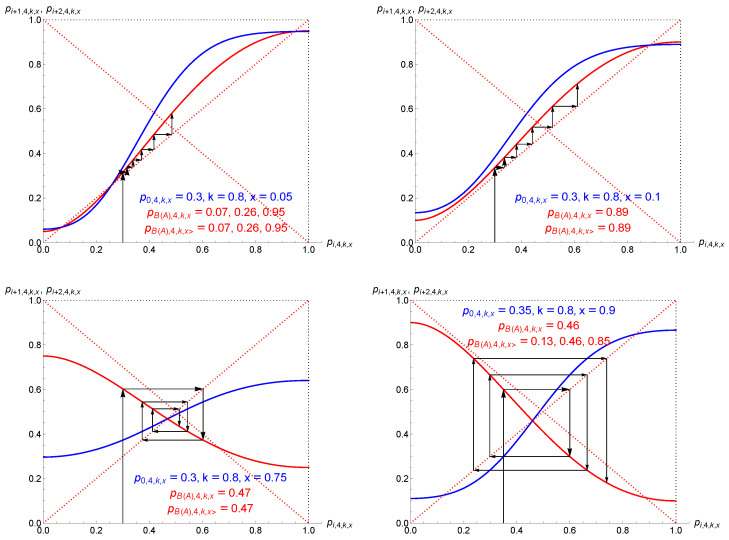
Four different regimes of the update $p_{i+1,4,k,x}$ are shown in red as a function of both *k* and *x*. The blue line shows $p_{i+2,4,k,x}$, which identifies the alternating attractors. The upper left part of the figure shows a tipping-point dynamics at a very low proportion of contrarians, with x=0.05 and k=0.80. The upper right part shows that already at x=0.10 the contrarians turn the dynamics to a single-attractor dynamics, with the attractor located at a very high value, with $p_{A,4,0.8,0.1}=0.89$. At high concentrations of contrarians, the dynamics stays single-attractor, as shown in the lower left part of the figure, with x=0.75 but with a lower value $p_{A,4,0.8,0.75}=0.47$. At x=0.85, the dynamics becomes alternating with two attractors, as exhibited in the lower right part of the figure.

Including contrarians and tie-breaking prejudice turns Equation (9) into

(18) $$p_{i+1,4,k,x}=\left(1-2x\right)\left\{p_{i,4,k,x}^{4}+4p_{i,4,k,x}^{3}\left(1-p_{i,4,k,x}\right)+6kp_{i,4,k,x}^{2}{\left(1-p_{i,4,k,x}\right)}^{2}\right\}+x,$$

whose fixed-point expressions are not reproduced here due to being very large analytical expressions. Moreover, the above results for $x>\frac{1}{2}$ indicate that the fixed-point equation $p_{i+2,4,k,x}=p_{i+1,4,k,x}$ must also be solved, i.e., a polynomial of degree 16. The associated fixed points would then be located by numerical solving. I thus identified four different regimes as a function of both *k* and *x*, as illustrated in Figure 6.

For very low values of the proportion *x* of contrarians, a tipping-point dynamics prevails, as seen in the upper left part of the figure. But already for x=0.10 a single-attractor dynamics is taking place, as seen in the upper right part of the figure.

When $x>\frac{1}{2}$, oscillations drive the dynamics, with two distinct regimes. A single-attractor regime takes place for $\frac{1}{2}<x<x_{c}$ (lower left part of figure) against an alternating-tipping-point dynamics for $x>x_{c}$ (lower right part of figure), where $x_{c}$ is very high, being larger or equal to 0.85.

**Figure 7:**
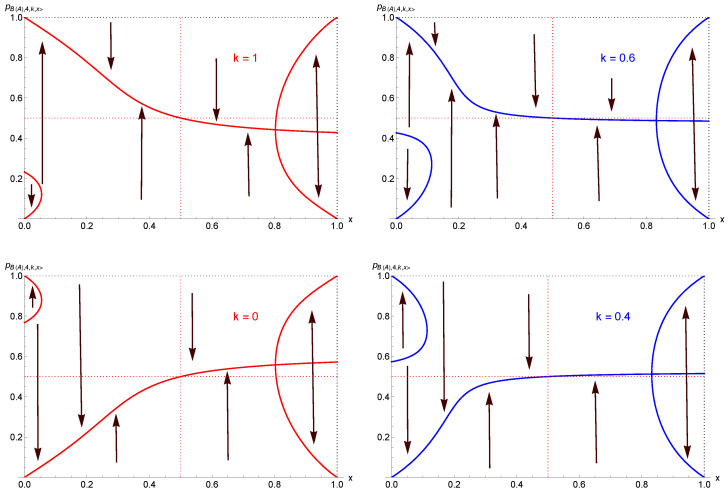
Evolution of attractors and tipping points as a function of the proportion *x* of contrarians for k=1 (**upper left part**), k=0 (**lower left part**), k=0.60 (**upper right part**), and k=0.40 (**lower right part**). Closed curves represent tipping points and attractors while single curves denote attractors except when inside a closed curve. Arrows indicate the direction of the flow of opinion dynamics while double arrows signal an alternating dynamics.

**Figure 8:**
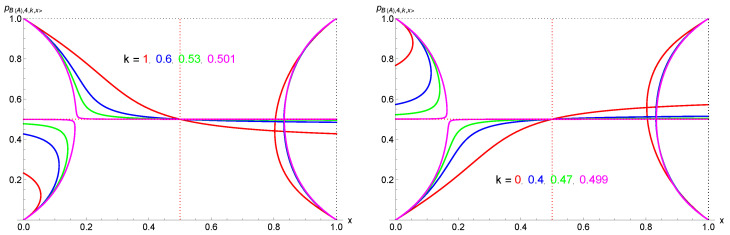

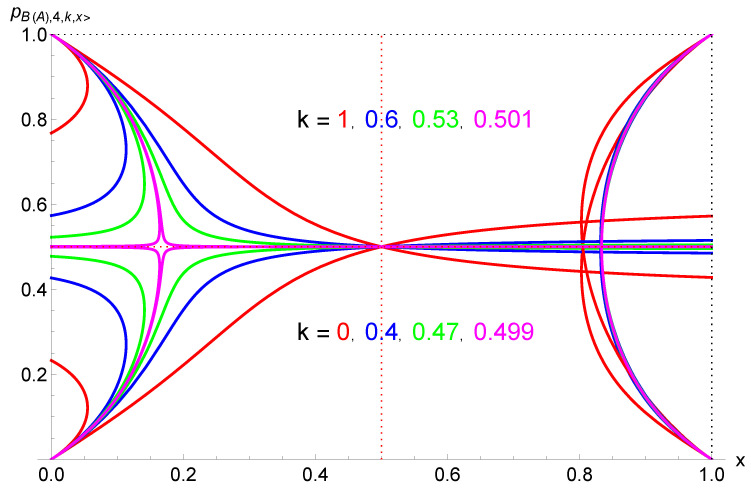
Evolution of attractors and tipping points as a function of the proportion *x* of contrarians for k=1,0.6,0.501 (upper left part) and k=0.499,0.4,0 (upper right part). Closed curves represent tipping points and attractors while single curves denote attractors except when inside a closed curve. The lower part combines both upper cases. The asymmetry between $k<\frac{1}{2}$ and $k>\frac{1}{2}$ as well as between $x<\frac{1}{2}$ and $x<\frac{1}{2}$ is clearly seen.

To build the complete landscape of the dynamics, I show in Figure 7 and Figure 8 the evolution of attractors and tipping points as a function of *x* for a given *k*. In the first figure, four cases illustrate the various types of landscape, with k=1,0.60,0.40,0. The second figure exhibits the combined cases k=1,0.60,0.53,0.501, k=0.499,0.47,0.40,0, and k=1,0.60,0.53,0.501,0.499,0.47,0.40,0. These figures shed light on the instrumental asymmetry between $k<\frac{1}{2}$ and $k>\frac{1}{2}$ as well as between $x<\frac{1}{2}$ and $x<\frac{1}{2}$. The part $x<\frac{1}{2}$ has been previously obtained [99].

## 5. A New Unexpected Regime of Stationary Alternating Polarization

The above results have uncovered the existence of a stationary alternating polarization at very high proportions of contrarians. The interplay with tie-breaking prejudices creates this new unexpected regime in the subspace $x>\frac{1}{2}$ for the three cases r=2,3,4. This discovery came as a surprise, with such a regime having been overlooked up to now.

For r=4, in the range $0\leq x<x_{c}$ the dynamics obeys a tipping-point regime, with values of $x_{c}$ being very low, as seen in Table 1. However, a proportion of contrarians in the range $x_{c}\leq x<\frac{1}{2}$ turns the tipping-point regime into a single-attractor regime.

When $\frac{1}{2}<x\leq x_{c>}$, with $x_{c>}$ being very large, as seen in Table 1, the dynamics stays within a single-attractor regime but now reaching the single attractor is achieved with an alternate dynamics.

Moreover, for $x>x_{c>}$, surprisingly, the dynamics turns back to a tipping-point regime but now with a stationary alternating regime between two attractors. The values of these attractors are given in Table 1. A symmetry between *k* and (1−k) is observed.

**Figure 9:**
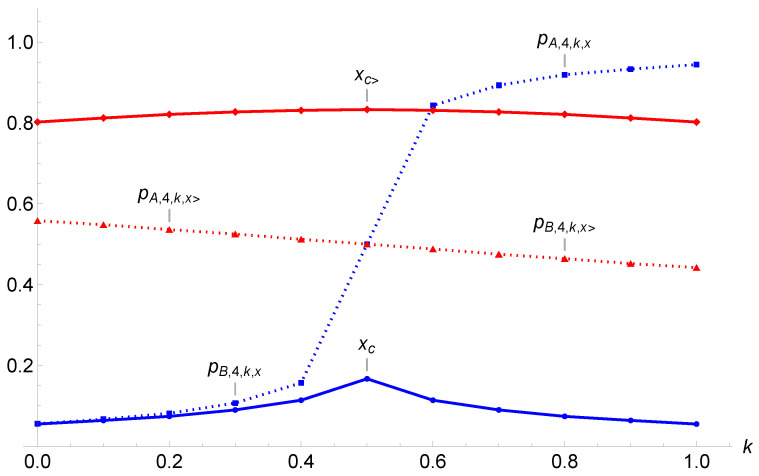
Plots of the values of $x_{c}$, $x_{c>}$, $p_{B,k\leq 0.5,x_{c}}$, $p_{A,k\geq 0.5,x_{c}}$, $p_{A,k\leq 0.5,x_{x>}}$, and $p_{B,k\geq 0.5,x_{x>}}$ as a function of *k* for r=4 according to the values of Table 1.

Figure 9 exhibits the values $x_{c}$, $x_{c>}$, $p_{B,k\leq 0.5,x_{c}}$, $p_{A,k\geq 0.5,x_{c}}$, $p_{A,k\leq 0.5,x_{x>}}$, and $p_{B,k\geq 0.5,x_{x>}}$ as a function of *k* according to the values of Table 1. It is worth noting that $x_{c>}\geq 0.802$ and $x_{c}\leq 0.167$.

Therefore, for any given values of *k* there exist two values of *x*, $x_{x}$ and $x_{x>}$, for which the regime is single-attractor-like.

It is noticeable that when $k<\frac{1}{2}$ opinion B always wins, with the single attractor $p_{B,k,x}$ located at low values, as seen in Figure 9. On the other hand, as soon as $k>\frac{1}{2}$ opinion A always wins, with $p_{A,k,x}$ located at high values. A jump occurs at $k=\frac{1}{2}$.

In contrast, opinion A always wins when $k<\frac{1}{2}$ but with a small margin, with $p_{A,k,x}$ slightly above $\frac{1}{2}$. When $k>\frac{1}{2}$, opinion B wins but with a small margin, with the attractor $p_{B,k,x}$ being located slightly below $\frac{1}{2}$. Moreover, there is no jump in the attractor values, with smooth changes as a function of *k*.

## 6. Conclusions

In this work, I have investigated the phenomenon of minority-to-majority transition in democratic public debates using the Galam Majority Model (GMM) of opinion dynamics. I thus built the full three-dimensional parameter space $\left(p_{0},k,x\right)$, where $p_{0}$ denotes the proportion of initial agents supporting opinion A, *k* is the probability of a local tie-breaking prejudice in favor of opinion A (the probability is (1−k) in favor of opinion B), and *x* is the proportion of contrarians. The individual traits associated with *k* and *x* are invisible to agents.

The focus was to identify the region in the subspace (k,x) where an initial $p_{0}<0.50$ ends up with $p_{n}>0.50$ after *n* successive updates of individual opinions. Three sizes r=2,3,4 were considered for local update groups. Tie-breaking prejudices apply only to even sizes in a tie, here r=2,4, and are absent for odd sizes like r=3.

For every given pair of values, *k* for tie-breaking prejudices and *x* for contrarians, the dynamics in *p* is driven by either a tipping point and two associated attractors or by a single attractor. A novel alternating polarization was also revealed at high proportions of contrarians.

**Tipping point:** In the first case, opinion A (B) needs to gather a proportion of initial support larger than the tipping point to ensure a democratic victory over time, with ongoing discussions among the agents. However, the results showed that this regime arises only for extreme proportions of contrarians, either very low or very high values. For r=2, one percent of contrarians suppresses the tipping-point regime.
**Alternating polarization:** A novel alternating-tipping-point regime was obtained at very high proportions of contrarians (92 percent or more) combined with very low and very high values of tie-breaking prejudices *k*. This regime is thus very extreme. For r=4, the tipping regime holds only for x<0.055 and x>0.802 at k=0,1, as seen from Table 1.
**Single attractor:** In the second case, which turned out to be the most common, the outcome of the dynamics is predetermined from the start, with a single attractor driving the dynamics of opinion. It is independent of the initial support $p_{0}$. Opinion A (B) cannot change the outcome, either victory or defeat depending on the current location of the single attractor with respect to 50%.

In summary, the results thus indicated that the expected democratic character of a free public debate is rarely satisfied. Indeed, most of the subspace (k,x) of the three-dimensional space $\left(p_{0},k,x\right)$ is governed by a single-attractor regime. Therefore, any initial support of opinions A and B ends up as the unique attractor, which is either above or below fifty percent for opinion A (B). The final outcome of a democratic public debate is thus predetermined independently of which opinion started as the majority in the related community.

On this basis, the only potential option for the supporters of the predetermined defeated opinion, would be trying to modify the actual values of (k,x) to reach a location, which is beneficial to that opinion in place of the other. Nevertheless, the challenge to identify and set the means to implement changes in *k* or *x* is out of the scope of the present paper.

### A Word of Caution

Last but not least, a word of caution is in order here: in case groundbreaking methods to actually change *k* or and *x* are pioneered, I am aware of the risk that my work could become an effective tool to manipulate opinion dynamics. However, the current situation, provided my model is sound, is shown to be embedded with invisible mechanisms, which are likely to “naturally” twist the democratic balance of a public debate in most cases. This highlights a kind of unconscious self-manipulation which skews the initial majority’s will. People can thus be taken to opposite wishes, leading to possible social and political disasters.

Striking and outstanding illustrations of such self-reversals of initial majorities have been both the case of the rejection of the French referendum to the proposal of a European constitution and Trump’s victory in the 2016 US presidential election. It is worth stressing that these two unexpected events were predicted successfully using the Galam Majority Model (GMM) [100,101].

I am convinced that only the discovery of the laws governing human behavior, in particular the dynamics of public opinion, can extract people, as a community, from being driven by their current ignorance and wrong beliefs into making inappropriate choices. In addition, once validated, a hard science implies both predicting and acting upon related phenomena. We are on a promising track with still a long way to go.

At this stage, my ethical responsibility as a scientist is to ensure that my results are freely accessible to everyone. The responsibility of the consequences of their use then lies among the future users, policy makers, and others.

## Figures and Tables

**Table 1:** Values of $x_{c}$ and $x_{c>}$ as a function of k=0, 0.1, 0.2, 0.3, 0.4, 0.5, 0.6, 0.7, 0.8, 0.9, 1 for r=4. The associated values $p_{B,k\leq 0.5,x_{c}}$, $p_{A,k\geq 0.5,x_{c}}$, $p_{A,k\leq 0.5,x_{x>}}$, and $p_{B,k\geq 0.5,x_{x>}}$ are also given. By symmetry, values are identical for *k* and (1−k).

k	0, 1	0.1, 0.9	0.2, 0.8	0.3, 0.7	0.4, 0.6	0.5
$x_{c}$	0.055	0.064	0.074	0.09	0.114	0.167
$p_{B,k\leq 0.5,x_{c}}$	0.056	0.067	0.0815	0.107	0.157	0.500
$p_{A,k\geq 0.5,x_{c}}$	0.944	0.933	0.919	0.893	0.843	0.500
$x_{c>}$	0.802	0.812	0.821	0.827	0.831	0.833
$p_{A,k\leq 0.5,x_{c>}}$	0.558	0.548	0.536	0.525	0.512	0.500
$p_{B,k\geq 0.5,x_{c>}}$	0.488	0.475	0.464	0.452	0.442	0.500

## Data Availability

Data is contained within the article.

## References

[1] https://www.ipsos.com/sites/default/files/ct/news/documents/2025-02/ipsos-cesi-la-tribune-les-francais-et-le-referendum-rapport-complet.pdf.

[2] Galam S. (2004). Contrarian Deterministic Effects on Opinion Dynamics: The Hung Elections Scenario. Phys. A.

[3] https://en.wikipedia.org/wiki/Condorcet_paradox.

[4] Brazil R. (2020). The physics of public opinion. Phys. World.

[5] Schweitzer F. (2018). Sociophysics. Phys. Today.

[6] Galam S. (2012). Sociophysics: A Physicist’s Modeling of Psycho-Political Phenomena.

[7] Chakrabarti B.K., Chakraborti A., Chatterjee A. (2006). Econophysics and Sociophysics: Trends and Perspectives.

[8] Galam S. (2022). Physicists, non physical topics, and interdisciplinarity. Front. Phys..

[9] da Luz M.G.E., Anteneodo C., Crokidakis N., Perc M. (2023). Sociophysics: Social collective behavior from the physics point of view. Chaos Solitons Fractals.

[10] Ellero A., Fasano G., Favaretto D. (2023). Mathematical Programming for the Dynamics of Opinion Diffusion. Physics.

[11] Oestereich A.L., Pires M.A., Queirós S.M.D., Crokidakis N. (2023). Phase Transition in the Galam’s Majority-Rule Model with Information-Mediated Independence. Physics.

[12] Filho E.A., Lima F.W., Alves T.F.A., de Alencar Alves G., Plascak J.A. (2023). Opinion Dynamics Systems via Biswas-Chatterjee-Sen Model on Solomon Networks. Physics.

[13] Zheng S., Jiang N., Li X., Xiao M., Chen Q. (2023). Faculty Hiring Network Reveals Possible Decision-Making Mechanism. Physics.

[14] Mobilia M. (2023). Polarization and Consensus in a Voter Model under Time-Fluctuating Influences. Physics.

[15] Li S., Zehmakan A.N. (2023). Graph-Based Generalization of Galam Model: Convergence Time and Influential Nodes. Physics.

[16] Malarz K., Masłyk T. (2023). Phase Diagram for Social Impact Theory in Initially Fully Differentiated Society. Physics.

[17] Kaufman M., Kaufman S., Diep H.T. (2024). Social Depolarization: Blume-Capel Model. Physics.

[18] Bittencourt R.A., Pereira H.B.B., Moret M.A., Galam S., Lima I.C.C. (2023). Interplay of self, epiphany, and positive actions in shaping individual careers. Phys. Rev. E.

[19] Zimmaro F., Olsson H. (2025). A meta-model of belief dynamics with Personal, Expressed and Social beliefs. arXiv.

[20] Zimmaro F., Galam S., Alberto Javarone M. (2024). Asymmetric games on networks: Mapping to ising models and bounded rationality. Chaos Solitons Fractals.

[21] Gimenez M.C., Reinaudi L., Galam S., Vazquez F. (2023). Contrarian majority rule model with external oscillating propaganda and individual inertias. Entropy.

[22] Aguilar-Janita M., Khalil N., Leyva I., Sendiǹa-Nadal I. (2025). Cooperation, satisfaction, and rationality in social games on complex networks with aspiration-driven players. arXiv.

[23] Kawahata Y. (2024). Theoretical Investigation of Harmful Information Propagation Using Physical Properties of Graphene and TeNPs: Sociophysical Approach. Biomed. J. Sci. Tech. Res..

[24] Bautista A. (2025). Opinion dynamics in bounded confidence models with manipulative agents: Moving the Overton window. arXiv.

[25] March-Pons D., Ferrero E.E., Miguel M.C. (2024). Consensus formation and relative stimulus perception in quality-sensitive, interdependent agent systems. Phys. Rev. Res..

[26] Shirzadi M., Zehmakan A.N. (2024). Do Stubborn Users Always Cause More Polarization and Disagreement? A Mathematical Study. arXiv.

[27] Li S., Phan T.V., Carlo L.D., Wang G., Do V.H., Mikhail E., Austinand R.H., Liu L. (2024). Memory and Personality Shape Ideological Polarization. arXiv.

[28] Demming A. (2023). The Laws of Division: Physicists Probe into the Polarization of Political Opinions. https://physicsworld.com/a/the-laws-of-division-physicists-probe-into-the-polarization-of-political-opinions/.

[29] Vilone D., Polizzi E. (2024). Modeling opinion misperception and the emergence of silence in online social system. PLoS ONE.

[30] Cui P.-B. (2023). Exploring the foundation of social diversity and coherence with a novel attraction-repulsion model framework. Phys. A.

[31] Liu W., Wang J., Wang F., Qi K., Di Z. (2024). The precursor of the critical transitions in majority vote model with the noise feedback from the vote layer. J. Stat. Mech..

[32] Banisch S., Shamon H. (2024). Validating argument-based opinion dynamics with survey experiments. The J. Artif. Soc. Soc. Simul..

[33] Pal R., Kumar A., Santhanam M.S. (2023). Depolarization of opinions on social networks through random nudges. Phys. Rev. E.

[34] Bagarello F. (2023). Phase transitions, KMS condition and decision making: An introductory model. Phil. Trans. R. Soc. A.

[35] Crokidakis N. (2023). Radicalization phenomena: Phase transitions, extinction processes and control of violent activities. Int. J. Mod. Phys. C.

[36] Coquidé C., Lages J., Shepelyansky D.L. (2023). Prospects of BRICS currency dominance in international trade. Appl. Netw. Sci..

[37] Mulyaa D.A., Muslim R. (2024). Phase transition and universality of the majority-rule model on complex networks. arXiv.

[38] Neirotti J., Caticha N. (2024). Rebellions and Impeachments in a Neural Network Society. Phys. Rev. E.

[39] Shen C., Guo H., Hu S., Shi L., Wang Z., Tanimoto J. (2023). How Committed Individuals Shape Social Dynamics: A Survey on Coordination Games and Social Dilemma Games. Eur. Phys. Lett..

[40] Grabisch M., Li F. (2020). Anti-conformism in the Threshold Model of Collective Behavior. Dyn. Games Appl..

[41] Forgerini F.L., Crokidakis N., Carvalho M.A.V. (2024). Directed propaganda in the majority-rule model. Int. J. Mod. Phys. C.

[42] Crokidakis N. (2024). Recent violent political extremist events in Brazil and epidemic modeling: The role of a SIS-like model on the understanding of spreading and control of radicalism. Int. J. Mod. Phys. C.

[43] Muslim R., Mulya D.A., Indrayani H., Wicaksana C.A., Rizki A. (2024). Independence role in the generalized Sznajd model. Phys. A Stat. Mech. Its Appl..

[44] Naumisa G.G., Samaniego-Steta F., del Castillo-Mussot M., Vázquez G.J. (2007). Three-body interactions in sociophysics and their role in coalition forming. Phys. A.

[45] Nettasinghe B., Percus A.G., Lerman K. (2025). How out-group animosity can shape partisan divisions: A model of affective polarization. PNAS Nexus.

[46] Hamann H. (2018). Opinion Dynamics With Mobile Agents: Contrarian Effects by Spatial Correlations. Front. Robot. AI.

[47] Guo L., Cheng Y., Luo Z. (2015). Opinion Dynamics with the Contrarian Deterministic Effect and Human Mobility on Lattice. Complexity.

[48] Toth G. (2024). Models of opinion dynamics with random parametrisation. J. Math. Phys..

[49] Tiwari M., Yang X., Sen S. (2021). Modeling the nonlinear effects of opinion kinematics in elections: A simple Ising model with random field based study. Phys. A.

[50] Chacoma A., Zanette D.H. (2014). Critical phenomena in the spreading of opinion consensus and disagreement. Physics.

[51] Cheon T., Morimoto J. (2016). Balancer effects in opinion dynamics. Phys. Lett. A.

[52] Landry N.W., Restrepo J.G. (2023). Opinion disparity in hypergraphs with community structure. Phys. Rev. E.

[53] Ahmad Mulyaa D., Muslim R. (2023). Destructive social noise effects on homogeneous and heterogeneous networks: Induced-phases in the majority-rule model. arXiv.

[54] Mihara A., Ferreira A.A., Martins A.C.R., Ferreira F.F. (2023). Critical exponents of master-node network model. Phys. Rev. E.

[55] Gártner B., Zehmakan A.N. (2020). Threshold Behavior of Democratic Opinion Dynamics. J. Stat. Phys..

[56] Chen P., Redner S. (2005). Majority rule dynamics in finite dimensions. Phys. Rev. E.

[57] Kułakowski K., Nawojczyk M. (2008). The Galam model of minority opinion spreading and the marriage gap. Int. J. Mod. Phys. C.

[58] Liu X., Achterberg M.A., Kooij R. (2024). Mean-field dynamics of the non-consensus opinion model. Appl. Netw. Sci..

[59] Ellero A., Sorato A., Fasano G. (2011). A New Model for Estimating the Probability of Information Spreading with Opinion Leaders. https://ssrn.com/abstract=2037450.

[60] Sendra N., Gwizdałła T., Czerbniak J. (2015). The application of cellular automata in modeling of opinion formation in society. Ann. Univ. Mariae-Curie-Sklodowska Sect. Ai–Inform..

[61] Crokidakis N., Blanco1 V.H., Anteneodo C. (2014). Impact of contrarians and intransigents in a kinetic model of opinion dynamics. Phys. Rev. E.

[62] Tòth G., Galam S. (2022). Deviations from the Majority: A Local Flip Model. Chaos Solitons Fractals.

[63] Kowalska-Styczeń A., Malarz K. (2020). Noise induced unanimity and disorder in opinion formation. PLoS ONE.

[64] Maksymov I.S., Pogrebna G. (2024). Quantum-Mechanical Modelling of Asymmetric Opinion Polarisation in Social Networks. arXiv.

[65] Redner S. (2019). Reality-inspired voter models: A mini-review. Comptes Rendus Phys..

[66] Jedrzejewski A., Marcjasz G., Nail P.R., Sznajd-Weron K. (2018). Think then act or act then think?. PLoS ONE.

[67] Singh P., Sreenivasan S., Szymanski B.K., Korniss G. (2016). Competing effects of social balance and influence. Phys. Rev. E.

[68] Bagnoli F., Rechtman R. (2015). Bifurcations in models of a society of reasonable contrarians and conformists. Phys. Rev. E.

[69] Carbone G., Giannoccaro I. (2015). Model of human collective decision-making in complex environments. Eur. Phys. J. B.

[70] Sznajd-Weron K., Szwabiński J., Weron R. (2014). Is the Person-Situation Debate Important for Agent-Based Modeling and Vice-Versa?. PLoS ONE.

[71] Javarone M.A. (2014). Networks strategies in election campaigns. J. Stat. Mech..

[72] Javarone M.A., Singh S.P. (2024). Strategy revision phase with payoff threshold in the public goods game. J. Stat. Mech..

[73] Goncalves S., Laguna M.F., Iglesias J.R. (2012). Why, when, and how fast innovations are adopted. Eur. Phys. J. B.

[74] Ellero A., Fasano G., Sorato A. (2009). A modified Galam’s model for word-of-mouth information exchange. Phys. A.

[75] Gimenez M.C., Reinaudi L., Vazquez F. (2022). Contrarian Voter Model under the Influence of an Oscillating Propaganda: Consensus, Bimodal Behavior and Stochastic Resonance. Entropy.

[76] Iacominia E., Vellucci P. (2023). Contrarian effect in opinion forming: Insights from Greta Thunberg phenomenon. J. Math. Sociol..

[77] Brugnoli E., Delmastro M. (2022). Dynamics of (mis)information flow and engaging power of narratives. arXiv.

[78] Sobkowicz P. (2020). Whither Now, Opinion Modelers?. Front. Phys..

[79] Weron T., Nyczka P., Szwabiński J. (2024). Composition of the Influence Group in the q-Voter Model and Its Impact on the Dynamics of Opinions. Entropy.

[80] Gsänger M., Hösel V., Mohamad-Klotzbach C., Müller J. (2024). Opinion models, data, and politics. arXiv.

[81] Vilela A.L.M., Zubillaga B.J., Wang C., Wang M., Du R., Stanley H.E. (2020). Three-State Majority-vote Model on Scale-Free Networks and the Unitary Relation for Critical Exponents. Sci. Rep..

[82] Mobilia M. (2011). Fixation and polarization in a three-species opinion dynamics model. Eur. Phys. Lett..

[83] Calvõ A.M., Ramos M., Anteneodo C. (2016). Role of the plurality rule in multiple choices. J. Stat. Mech..

[84] Maciel M.V., Martins A.C.R. (2020). Ideologically motivated biases in a multiple issues opinion model. Phys. A.

[85] Dworak M., Malarz K. (2023). Vanishing opinions in Latané model of opinion formation. Entropy.

[86] Ferri I., Gaya-Ávila A., Díaz Guilera A. (2023). Three-state opinion model with mobile agents. Chaos.

[87] Oliveiraa I.V.G., Wang C., Dong G., Du R., Fiore C.E., Stanley H.E., Vilela A.L.M. (2023). Entropy Production on Cooperative Opinion Dynamics. arXiv.

[88] Coquidé C., Lages J., Shepelyansky D.L. (2024). Opinion Formation in the World Trade Network. Entropy.

[89] Timpanaro A.M. (2025). Emergence of echo chambers in a noisy adaptive voter model. arXiv.

[90] Ye D., Lin H., Jiang H., Du L., Li H., Chen Q., Wang Y., Yuan L. (2025). Simplex bounded confidence model for opinion fusion and evolution in higher-order interaction. Expert Syst. Appl..

[91] Deffuant G. (2024). Complex Transitions of the Bounded Confidence Model from an Odd Number of Clusters to the Next. Physics.

[92] Malarz K., Gronek P., Kulakowski K. (2011). Zaller-Deffuant Model of Mass Opinion. J. Artif. Soc. Soc. Simul..

[93] Galam S. (1990). Social paradoxes of majority rule voting and renormalization group. J. Stat. Phys..

[94] Galam S., Chopard B., Masselot A., Droz M. (1998). Competing species dynamics: Qualitative advantage versus geography. Eur. Phys. J. B.

[95] Galam S. (2002). Minority Opinion Spreading in Random Geometry. Eur. Phys. J. B.

[96] Galam S., Moscovici S. (1991). Towards a theory of collective phenomena: Consensus and attitude changes in groups. Eur. J. Soc. Psychol..

[97] Galam S., Jacobs F. (2007). The role of inflexible minorities in the breaking of democratic opinion dynamics. Phys. A.

[98] Galam S. (2023). Unanimity, Coexistence, and Rigidity: Three Sides of Polarization. Entropy.

[99] Galam S. (2024). Fake News: “No Ban, No Spread—With Sequestration”. Physics.

[100] Le Hir P. Les Mathématiques S’invitent Dans le Débat Européen, Le Monde. Le Monde, 25 February 2005. https://www.lemonde.fr/planete/article/2005/02/25/les-mathematiques-s-invitent-dans-le-debat-europeen_399570_3244.html.

[101] Galam S. (2017). The Trump phenomenon: An explanation from sociophysics. Int. J. Mod. Phys. B.

